# Gender bias in machine learning: insights from official labour statistics and textual analysis

**DOI:** 10.1007/s11135-025-02261-0

**Published:** 2025-07-08

**Authors:** Orfeas Menis–Mastromichalakis, George Filandrianos, Maria Symeonaki, Glykeria Stamatopoulou, Dimitris Parsanoglou, Giorgos Stamou

**Affiliations:** 1 School of Electrical and Computer Engineering, National Technical University of Athens, Athens, Greece; 2 Department of Social Policy, Panteion University of Social and Political Sciences, Athens, Greece; 3 Department of Sociology, National and Kapodistrian University of Athens, Athens, Greece

**Keywords:** Gender occupational segregation, EU-LFS, ISCO-08, Machine learning, Automatic translation bias

## Abstract

The interplay between technology and societal norms often reveals a troubling reality: machine learning systems not only reflect existing gender stereotypes but can also amplify and entrench them, making these biases harder to detect and address. This paper adopts an interdisciplinary approach, combining quantitative and qualitative methods with recent technological advancements, such as machine learning techniques for textual analysis and computational linguistics, to offer a new framework for understanding occupational gender bias in machine learning. The study is motivated by persistent gender inequalities in the labour market and rising concerns about gendered algorithmic bias, as outlined in the European Commission’s Gender Equality Strategy 2020–2025. Focusing on language translation technologies, the research explores how machine learning may perpetuate or amplify gender stereotypes, aiming to foster more inclusive digital systems aligned with EU strategic goals. More specifically, it investigates occupational gender segregation and its manifestations in various forms of gender bias in machine learning across English, French, and Greek. The study introduces a classification of gender biases in machine learning, providing insights into professional areas needing intervention to address gender imbalances and identifying enduring stereotypical representations in textual data. To support this, statistical analysis is conducted to explore gender variations in occupations over the past thirteen years, using official data and international classifications such as the International Standard Classification of Occupations (ISCO-08). Moreover, gendered occupational distributions are extracted from 200,920 text instances in the three languages, revealing significant discrepancies between official labour statistics and the training data.

## Introduction

Academic literature clearly shows a stable, growing trend in using comparative multimethod approaches in social policy research, planning and implementation. The study of social policy research methods has garnered significant attention and has during the last years shown an increasing trend in mixed methods particularly pronounced in Sociology (Ferragina and Deeming 2023). In this paper, we employ an exemplary synthesis of quantitative and qualitative methodologies, along with recent Artificial Intelligence (AI) technological advancements, i.e. methods for examining textual data and computational linguistics, to offer an innovative approach in understanding gender bias in automated translation systems.

Gender stereotypes are pervasive and deeply rooted social beliefs about the traits and roles of men and women, which often shape expectations and behaviours in subtle but powerful ways. Even decades ago, these stereotypes were commonly divided into two overarching categories: agency, typically linked to men (e.g., assertiveness, independence, ambition), and communion, generally associated with women (e.g., nurturing, kindness, emotional expressiveness) (Eagly and Karau 2002; Broverman et al. 1972). In recent years, researchers have begun to adopt a more refined perspective on agency and communality, breaking each down into distinct components to better identify which specific traits are more closely linked to one gender over the other (Heilman et al. 2024).

The existence of stereotypes can be explained as serving certain cognitive functions, such as simplifying complex social information (Macrae et al. 1994). Nevertheless, they result in biased generalisations, especially when applied to individuals (Agars 2004; Welle and Heilman 2007). In the case of gender stereotypes, they are both descriptive (how men and women are perceived to be) and prescriptive (how they are expected to behave), contributing to systemic gender inequalities (Heilman 2012; Eagly 1987).

Occupational roles are also strongly gendered, with people consistently perceiving certain jobs as more suitable for men or for women, regardless of other parameters, such as age, educational background or qualification (Gottfredson 1981; Glick et al. 1995). One of the most common and researched forms of gender disparity is that of the gender pay gap, which is mainly due to the horizontal and vertical occupational segregation that this paper addresses (Carlsen et al. 2023). According to the results of the present study based on raw data from the EU Labour Force Survey (EU-LFS), significant gender disparities persist in occupational distributions across EU countries. For instance, in 2023, women remained underrepresented in several high-status professions, e.g. in the ICT sector, while dominating others such as nursing and teaching. Additionally, both horizontal and vertical segregation persist as significant issues, with horizontal segregation reflecting disparities in the gender concentration across different sectors and occupations, and vertical segregation indicating unequal distributions across grades, levels of responsibility, and positions within specific sectors (EU Science Hub 2024; EIGE 2024). The findings from twenty selected high-income OECD countries, presented in Khan et al. (2017), indicate that significant policy reforms in the labour market are needed to address persistent inequalities, which impede long-term economic growth.

From a technological point of view, it is widely acknowledged that large language models (LLMs) tend to inherit and amplify societal biases present in their training data, which can inadvertently reinforce harmful stereotypes related to gender, occupation, and other sensitive categories (Gorti et al. 2024). Recent studies have increasingly concentrated on detecting and addressing gender biases in machine translation systems. For instance, research by Marcelo et al. (2020) has empirically shown that commercial translation tools frequently reinforce societal gender stereotypes by incorrectly assigning genders to professions rather than adhering to linguistic accuracy. Likewise, Zhao et al. (2017) emphasised the bias of translation algorithms toward masculine pronouns, even in cases where the gender is not specified. In the realm of LLMs, Ghosh and Caliskan (2023) highlighted that systems, such as ChatGPT, Google Translate, and Microsoft Translator, tend to reinforce gender norms and defaults, particularly struggling to translate the English gender-neutral pronoun “they” into equivalent gender-neutral pronouns in other languages. This often results in inaccurate and incoherent translations, especially for languages with limited resources.

Several other studies have explored biases in natural language processing (NLP) systems, particularly examining the stereotypical associations between gender and specific occupations. Bolukbasi et al. (2016) highlighted how word embeddings, a foundational component of many NLP systems, often reflect and perpetuate societal biases, including those linking certain professions to specific genders. Caliskan et al. (2017) showed that semantics derived automatically from language corpora contain human-like biases. Salinas et al. (2023) expanded on this by investigating how contemporary machine learning models inherit and sometimes amplify these biases, with a focus on practical implications in occupational contexts. Similarly, Sheng et al. (2019) examined the generation of biassed outputs in NLP systems, emphasising the role of stereotypes in shaping the portrayal of gendered professions. A recent study by Ducel et al. (2024) introduced a framework for automatically evaluating gender bias in language models, analysing 52,000 French cover letters and 4,100 Italian ones. In this study, the gender gap is defined as the difference in the proportions of documents classified as masculine versus feminine. This metric is regarded as a significant indicator of gender bias, with a model considered biassed if it produces uneven distributions of masculine and feminine outputs. Our study adds to the existing literature by analysing actual gendered occupational distributions and evaluating the discrepancies between actual occupational data and information derived from textual datasets. This analysis is conducted through experiments in three languages, utilising over 200,000 text instances from two major training datasets, namely WMT (Bojar et al. 2014) and the Colossal Clean Crawled Corpus (C4) (Raffel et al. 2020). In a similar vein, Gorti et al. (2024) examined how the detected bias in LLMs correlates with authoritative datasets, such as those drawn from the U.S. National Bureau of Labour Statistics.

The methodology proposed and used in this study is designed to investigate occupational gender segregation and how this phenomenon manifests in various types of gender bias in machine translation, in English, French, and lower-resource languages like Greek. These languages were selected because they represent diverse linguistic structures, ranging from notional-gender languages (English) to languages with grammatical gender (French and Greek), allowing us to explore how grammatical gender interacts with gender bias in occupational language. An additional rationale lies in the linguistic diversity offered by the inclusion of French and Greek, which belong to different language families, i.e. Romance and Hellenic, respectively. This diversity enhances the value of comparative analysis, particularly given that English, the source language in this context, is the most widely used language in NLP and machine translation (MT). Furthermore, these countries offer reliable microdata and sufficient volumes of text for analysis, making them suitable for cross-linguistic and cross-national comparison.

A framework for classifying gender bias in machine learning is proposed, offering valuable insights into long-established and persistent gender stereotypes in the workforce. This classification serves a dual purpose: first, to identify professional sectors where further action is needed to address chronic gender imbalances; and second, to detect recurring stereotypical representations that persist despite data showing a different reality. Thus, our motivation derives from two primary concerns: the persistent gender inequalities in the labour market, and the existence of gendered algorithmic bias (Gorti et al. 2024; Kirk et al. 2021; Thakur 2023; Vanmassenhove 2024), as they are both highlighted in strategic social policy documents such as the European Commission’s Gender Equality Strategy 2020–2025 (European Commission 2020). More specifically, in 2020 the European Commission adopted this strategy, which outlines key actions for the five-year period and commits to incorporating an equality perspective across all EU policy areas. In its current work program, the Commission has reaffirmed its dedication to promoting gender equality, with a focus on thematic priority areas such as eliminating violence and stereotypes and closing gender gaps in the labour market. The Strategy emphasises the necessity of challenging gender stereotypes, which are fundamental drivers of gender inequality across all societal domains. It emphasises the importance of achieving equal representation across economic sectors, noting that although women outnumber men among university graduates in Europe, they remain underrepresented in higher-paying professions.

Moreover, the Strategy places specific focus on the impact of AI, highlighting the need for further exploration of its potential to amplify or contribute to gender biases. Specifically, gender bias in machine translation systems is underlined as a significant element of this aspect. Here, by utilising labour statistical data to assess the presence of stereotypical automatic translations, we can identify instances where these systems perpetuate or escalate gender stereotypes in the labour force. This inquiry helps examine whether language translation, and machine learning technologies in general, accurately reflect and respect gender occupational diversity. Addressing gender bias in machine learning is essential for creating inclusive and equitable communication platforms that uphold the principles of diversity and non-discrimination advocated by the European Commission’s Gender Equality Strategy. Gender bias in translation is also evident in various instances and across a broad spectrum of formal publications. For example, in the Greek translation of the European Union Labour Force Survey (EU-LFS) questionnaire, the response category for the question regarding the respondent’s main current activity translates “Fulfilling domestic tasks” as “*She* is doing domestic work,”^1^ implying that domestic tasks are exclusively performed by women.

The aim of this study is to investigate the interconnected dynamics between gender stereotypes, labour market gender inequalities, and gender bias in machine learning systems, with a particular focus on linguistic encoding of occupational gender and automated translation technologies. Decades of research have shown that gendered occupational associations form early in life; children as young as three already link certain professions to specific genders, e.g. nurses with women and doctors with men- (Blakemore 2003; Fox and Barth 2017). These stereotypes not only persist into adulthood but are also embedded in digital technologies. For instance, automated translation systems translate gender-neutral phrases like “the doctor” as male and “the nurse” as female. However, such representations often contradict real-world data: our analysis for example reveals that, at the European level, women now outnumber men in the medical profession. This highlights a misalignment between labour market realities and the gendered assumptions encoded in AI-driven language tools. Acknowledging that individuals adapt to and perpetuate stereotypical gender roles as they grow within cultural and social environments (Corcoran and Courant 1985; Crompton and Harris 1998; England et al. 1994; Gaskell 1983; Greenough et al. 1987; OECD 2023; European Commission 2010; Symeonaki and Filopoulou 2017; Symeonaki et al. 2019), we conduct an in-depth analysis of gender occupational segregation, the stereotypical conceptualisation of occupations, the representation of occupational roles in text, and the ways in which automated translation systems may contribute to the perpetuation of gender bias. In a more specific setting, De Gioannis (2024) explored the causes of gender-science stereotypes, categorising them into external factors (such as discrimination) and internal factors (such as ability and interest), aligning with the attribution of stereotypes theory proposed by Reyna (2000). These stereotypes remain remarkably stable over time, even as explicit beliefs about gender roles become less rigid. Large-scale studies, such as Nosek et al. (2009), demonstrate persistent implicit associations linking men with science and leadership, and women with caregiving and supportive roles -associations that are often unconscious but nonetheless influential. More recent studies reaffirm that gender stereotypes continue to shape career aspirations, hiring decisions, and evaluations of competence, especially when there is a perceived lack of fit between gender and job role (Heilman 2001; Eagly et al. 2020).

Our study builds on previous analysis by proposing an additional external factor for persisting stereotypes: the gender bias inherent in contemporary machine learning systems, which are increasingly prevalent in various applications. To achieve our objective, we perform statistical analyses on raw microdata drwan from the European Union Labour Force Survey (EU-LFS) to trace gender variations in occupational distributions and their evolution over the past thirteen years. For France and Greece, occupations are classified according to the International Standard Classification of Occupations (ISCO-08) at the 3-digit level over the period 2011–2023. For the United Kingdom, we use the ISCO-08 classification for the years 2011–2019 (EU-LFS data) and the Standard Occupational Classification (SOC 2020) for the post-Brexit period (Official statistics). In addition, our methodology includes the construction of a curated Knowledge Graph (KG) that:

1. Encodes standardised knowledge based on international occupational classifications,
2. Utilises gendered comparative statistical analysis on occupational data drawn from large-scale databases such as the EU-LFS, and
3. Incorporates statistical measurement for occupation-related gendered language usage derived from linguistic corpora.

Knowledge Graphs (KGs) have seen growing use in advancing responsible and equitable AI systems (Pan et al. 2023). In addition, Mehrabi et al. (2021) present an extensive review of bias in AI, underscoring the importance of KGs in identifying and mitigating biases. Their study illustrates how incorporating KGs into machine learning models can improve the transparency and accountability of AI applications. The KG developed in the present study can be used to classify gender misalignment in machine translation or any other machine-generated gender misalignment (e.g. erroneous gender assignment in generative models) into three categories of potentially harmful gender bias (Savoldi et al. 2021):

1. *Under-representational bias* occurs when gender assignment aligns with an imbalance in linguistic corpora which reflects the low representation of a specific gender in sociodemographic reality. For example, “the plumber” is typically translated as masculine, which aligns with the fact that women are very rarely plumbers.
2. *Stereotypical bias* occurs when gender assignment aligns with negative generalisations resulting from an imbalance in linguistic corpora, rather than reflecting social reality. For example, “the doctor” may be translated as masculine due to its predominant use as masculine in linguistic corpora, despite the equal possibility of doctors being women.
3. *Algorithmic bias*, when the gender assignment is possibly due to a machine learning algorithmic failure (not grounded to a low representation of a specific gender in linguistic corpora).

The proposed combination of methods and resources will enable the study of both longitudinal occupational gender segregation across these European countries, offering a comprehensive analysis of gender disparities in various occupational sectors. The findings from this analysis will inform the development of actionable policy recommendations aimed at reducing gender segregation. Additionally, this approach can contribute to the creation of guidelines for unbiased language translation and text generation, addressing the presence of gender bias in machine learning systems. By raising awareness of these biases, the study aims to promote more equitable and inclusive language practices in both social policy and AI technology.

The paper is structured as follows. Section 2 provides a detailed overview of the data sources and methodology employed, emphasising both the statistical analyses performed, and the techniques used for extracting gender statistics from datasets and creating the knowledge graph. Section 3 explores patterns of occupational gender segregation in the countries under analysis and evaluates how these align with gendered occupational representations found in the text corpora. Section 4 identifies key limitations of the study, whereas the concluding section summarises the significant insights from the analysis and offers recommendations for future research, including potential improvements in bias detection methods and broader applications of the findings.

## Materials and methods

This study adopts a mixed-methods approach that integrates quantitative labour market data with large-scale textual analysis to investigate how occupational gender segregation is reflected and potentially reinforced in language use and by extension to automated translation. Drawing on raw microdata from the EU Labour Force Survey (EU-LFS), we analyse gender distributions across occupations in three countries, France, Greece, and the UK, over time. The Duncan index of dissimilarity (Duncan and Duncan 1955) is used to estimate and compare gender occupational segregation across the three studied countries and over the years (2011–2023). In parallel, we extract and annotate gendered occupational references from a corpus of over 200,000 texts across English, French, and Greek. To assess how gendered occupational language is handled by automated systems, we use typical examples of textual data and their translation using widely used machine translation tools, such as Google Translate and DeepL, enabling us to examine the extent to which stereotypical gender associations are preserved or introduced in translation and how divergent the actual occupational statistics are from the textual ones. The following sections detail our data sources, processing steps, and analytical methods.

### Data and statistical analysis on gender occupational segregation

The present paper performs a statistical analysis on raw microdata drawn from the European Union Labour Force Survey (EU-LFS), for the years 2011–2023, provided by EUROSTAT for scientific purposes. The EU-LFS is the largest European sample survey offering quarterly and annual statistics on labour market participation and inactivity for individuals over 15 years old (EUROSTAT 2024). It also serves as the main data source for European statistics due to its expansive range of questions and sample size, enabling comparability across countries and over time through standardised definitions, classifications, and variables. EU-LFS spans residents in private households across thirty-five participating countries, giving valuable information to EUROSTAT, which is responsible for centrally processing the data. More specifically, the survey is conducted in all EU countries, four candidate countries, and three European Free Trade Association (EFTA) countries. The most common sample design for the EU-LFS is stratified two-stage cluster sampling, with stratified one-stage cluster sampling also in use. The International Labour Organisation (ILO) guidelines are followed and standard classifications such as the International Standard Classification of Occupations (ISCO-08), the International Standard Classification of Education (ISCED), and the nomenclature of territorial units for statistics (NUTS) allow for comparability among countries. More specifically, EU-LFS uses ISCO-08 at 4-digit level for the current main job and at 3-digit level for the last job. However, EUROSTAT provides the microdata about employed persons and their main job only at ISCO-08 3-digit level. Additional secondary analysis is performed from the NOMIS (UK Official Census and Labour Market Statistics) to cover for the UK for the years 2019–2023. More specifically, the analysis presented in the study covers France and Greece from 2011 to 2023 at the ISCO-08 3-digit level, the UK from 2011 to 2019 at the ISCO-08 3-digit level, and from 2020 to 2023 the SOC 2020 classification at 3- and 4-digit level. The objective of this analysis is to estimate the distribution of males and females across occupations at the most detailed level available for the UK, France, and Greece over the past thirteen years.

Moreover, we calculate the Duncan index of dissimilarity, also known as the Duncan segregation index (Duncan and Duncan 1955), a widely used measure of occupational gender segregation. This index quantifies the extent to which the distribution of men and women across occupations deviates from equality by estimating the proportion of either group that would need to change occupations for gender parity to be achieved. More specifically, we compute the average occupational segregation index across all occupations at the 3-digit ISCO-08 level, for the three countries over the period 2011 to 2023, by first computing the segregation index for each occupation:

1 $${D}_{i}=\frac{1}{2}\left|\frac{{f}_{i}}{{f}_{i}+{m}_{i}}-\frac{{m}_{i}}{{f}_{i}+{m}_{i}}\right|$$

where ${f}_{i}, {m}_{i}$ is the number of women and men in occupation $i=\mathrm{1,2},\dots ,n.$ We then compute the total dissimilarity index for each country by averaging the ${D}_{i}$’s:

2 $$D=\frac{{\sum }_{i=1}^{n}{D}_{i}}{n}.$$

To assess potential gender bias in occupational representation, we compared male-to-female ratios in actual labour force statistics with those observed in textual references across major ISCO-08 occupational groups. For each group, we constructed 2 × 2 contingency tables using male and female counts from both sources and applied Fisher’s exact test to evaluate whether the gender distribution in the text differed significantly from the official data. This test was chosen for its accuracy with categorical data and small sample sizes. We report odds ratios to indicate the direction and magnitude of disparities, and corresponding p-values to assess statistical significance. All analyses were conducted using SPSS 29.1.0 and Python 3.13.

#### The international classification of occupations (ISCO)

The classification of occupations aims to support global discourse on occupations by providing labour statisticians and in general researchers addressing issues related to employment with a structure to extract internationally comparable occupational data. This section provides the reader with details regarding occupational classifications presently utilised within the field.

The ISCO framework introduced by the International Labour Organisation (ILO) enables the production of international occupational data in formats suitable for research and actionable initiatives. It is a four-level hierarchically structured classification designed to offer a comprehensive framework for categorising all jobs worldwide. Most countries utilise this classification when reporting labour statistics to the United Nations Statistics Division, being also the standard adopted by EUROSTAT for reporting purposes. From 2011 and onwards the EU-LFS uses the ISCO-08 classification of occupations (EU Labour Force Survey Database User Guide 2024: p. 94). According to the official description of the ISCO-08 taxonomy, the major groups (1-digit) are (ILO 2012: p. 65; ILO 2023a: p. 14):

- 0-Armed Forces Occupations,
- 1-Managers,
- 2-Professionals,
- 3-Technicians and Associate Professionals,
- 4-Clerical Support Workers,
- 5-Service and Sales Workers,
- 6-Skilled Agricultural, Forestry and Fishery Workers,
- 7-Craft and Related Trades Workers,
- 8-Plant and Machine Operators, and Assemblers,
- 9-Elementary Occupations.

Examining the hierarchical structure of ISCO-08 from the highest level downwards, the ten major groups consist of one or multiple sub-major groups, which, in turn, comprise one or several minor groups (totalling 130 minor groups). Each group within the classification is identified by a title and code number, accompanied by a description delineating the group’s scope. More specifically, Table 6 in the Appendix presents the number of groups at each level and the 10 major groups of the ISCO-08 classification.^2^

- Major Group is denoted by a 1-digit code (10 in total),
- Sub-Major Group is denoted by a 2-digit code (43 in total),
- Minor Groups are denoted by 3-digit codes (130 in total), and
- Unit Groups are denoted by 4-digit codes (436 in total).

In the UK, up until 2019, the ISCO-08 classification was employed as a requirement to align with the taxonomy used in the EU-LFS, while concurrently, the UK has maintained the use of the Standard Occupational Classification for the UK (SOC 2020). More information related to this classification is provided in the Appendix.

### Extracting gender statistics from textual datasets

**Fig. 1:**
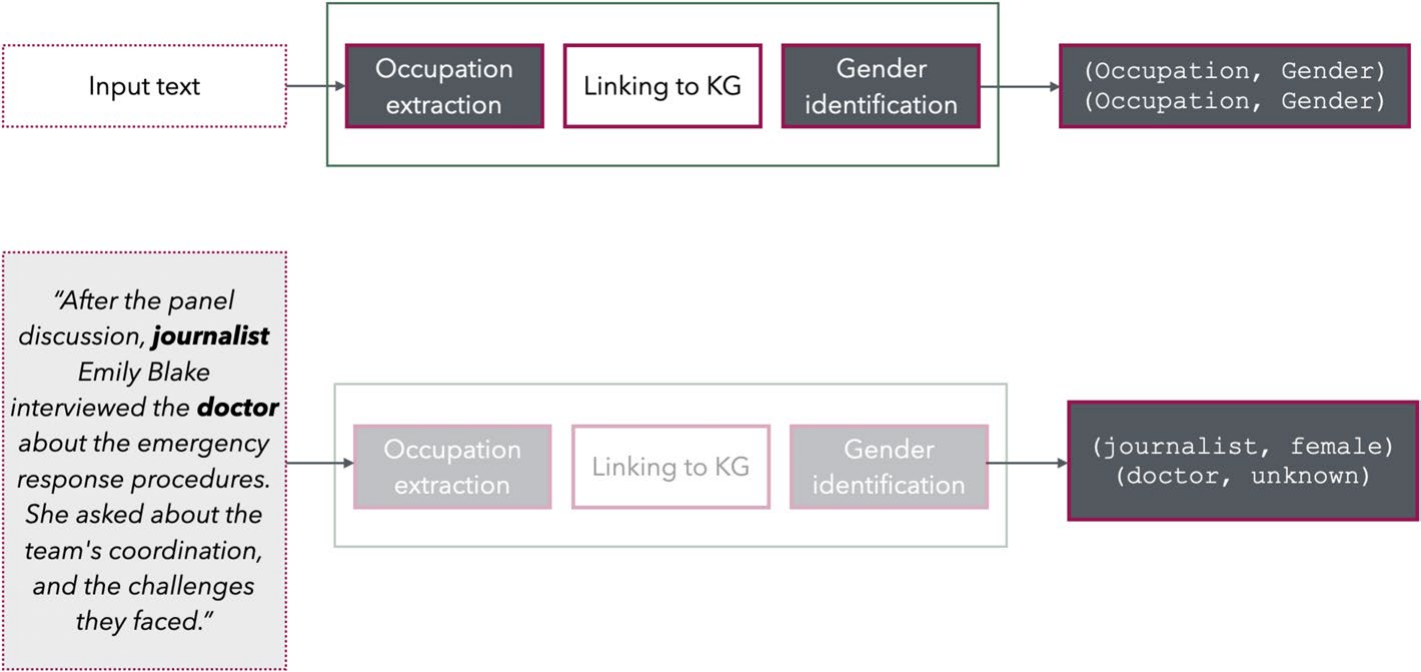
Pipeline for extracting and analysing gender statistics from textual datasets for the KG and an input–output example

The proposed methodology for extracting gender statistics from textual datasets involves a multi-stage pipeline designed to identify occupations, link them to the Knowledge Graph (KG), and determine their associated gender references. This specific process consists of three main stages: Occupation Extraction, Linking to the KG, and Gender Identification. Each stage is critical for ensuring accurate and reliable analysis of gender representation in occupations. Figure 1 describes the pipeline for extracting and analysing gender statistics from textual datasets and offers an input–output example. Technical information concerning this multi-stage procedure can be found in the Appendix.

The first step in this process is to identify occupations mentioned within the text. To achieve this, we use a Large Language Model (LLM) and apply a technique known as *zero-shot prompting*, meaning that we instruct the LLM to perform the task without prior examples. The model scans the text to detect occupational terms based on their surrounding context and generates brief descriptions for each identified role. These descriptions help group together occupational terms that refer to the same job, by linking each detection to the ISCO-based Knowledge Graph, accounting for variety in wording or phrasing. They also help reduce the risk of *hallucinations*, a common issue in large language models where the system generates information that sounds plausible but is factually incorrect, made-up, or not grounded in reality.

Following the identification of occupations, each detected term is subsequently linked to a corresponding ISCO-08 classification code. This step facilitates the grouping of similar occupations and enables consistent comparison across datasets. The linking process involves comparing the generated textual descriptions of the extracted occupations to official definitions contained within a structured resource, the Knowledge Graph. Both the model-generated and official descriptions are transformed into a mathematical format (called *vector embeddings*), that allows us to measure how similar they are. Matches are determined based on a similarity score, with a predefined threshold applied to filter out weak or incorrect associations and hallucinations. This approach mitigates potential errors from the detection phase and ensures that only standardised and valid occupational categories are incorporated into the analysis.

The final and most complex step in the pipeline is the Gender Identification module, which assigns gender to each detected occupation using a three-step process. First, in languages with grammatical gender (e.g., Greek), the occupation term itself may indicate gender and is identified using tools like *SpaCy*. Second, if the occupation is gender-neutral, associated pronouns (e.g., “*He* is a *teacher*”) are used to infer gender through syntactic dependency analysis. Third, when neither of the above is present, coreference resolution (e.g. “The *teacher* left the school in a hurry. It was only later that *he* realised that *his* bag was still at school.”) is applied to infer gender from broader context using the *Coreferee* library. If no reliable gender reference is found, the occupation is excluded from the gender statistics. Successfully identified genders are then integrated into the Knowledge Graph, enriching it with gender-specific occupational data drawn from text.

### Integrating gender statistics into a knowledge graph

**Fig. 2:**
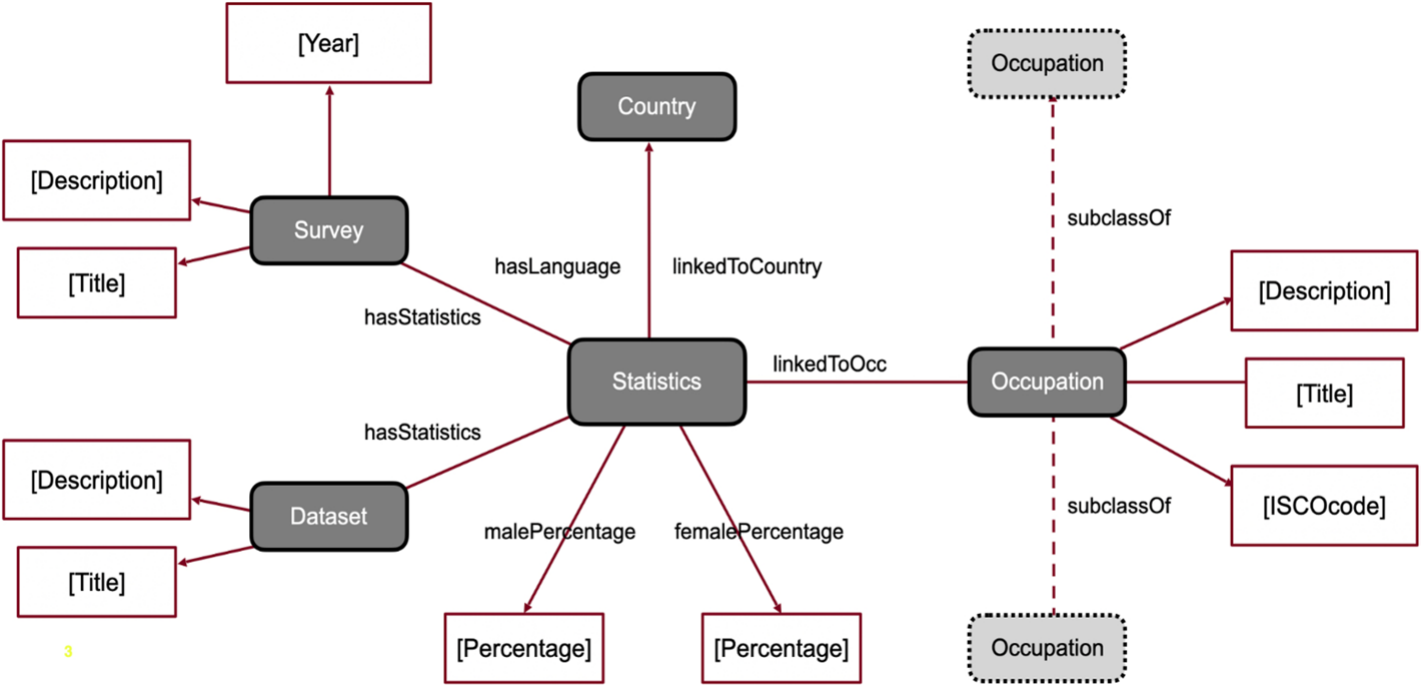
The knowledge graph’s structure

To incorporate the extracted statistics into a structured format we built a knowledge graph (KG), i.e. a structured representation of information that captures relationships between different entities in a graph format, designed to integrate, manage, and retrieve information from diverse sources, enabling a richer understanding of the connections and context within the data (Hogan et al. 2021; Noy et al. 2019). The Knowledge Graph developed in the current study uses the hierarchy classification of occupations based on ISCO-08 to encode standardised knowledge and data relating to occupations. This hierarchy organises occupations in the KG, following the same categorisation as the ISCO-08 taxonomy, into major groups (1-digit), subgroups (2-digits), minor (3-digits) and unit groups (4-digits) linked through the “subclassOf” ‘relations. For instance, the major group “Managers” (ISCO-08 code 1) includes “Chief Executives, senior officials and legislators” (code 11) as a subclass, which further branches into several occupations including “Legislators and senior officials” (code 111) and “Managing directors and chief executives” (code 112). Each occupation in the KG has a title, e.g. “Medical doctors”, a description^3^ and an ISCO-08 code (e.g. 221), all extracted from the ISCO-08 classification. It also makes use of the statistical results described in Sect. 3.1 based on the EU-LFS and NOMIS -the UK Official Census and Labour Market Statistics. It further incorporates statistics for occupation-related gendered language usage derived from linguistic corpora. Statistical data is further linked with a country, a specific survey and the year the survey was conducted, or a dataset for which a description and a title are given. Figure 2 illustrates the structure of the developed Knowledge Graph.

### Classifying gender bias

Using the above Knowledge Graph, we can classify gender bias based on real-world and dataset statistics, indicating possible sources of bias. When gender bias is detected, it can be classified as under-representational when the gender assignment reflects the low representation of a specific gender in linguistic corpora, consistent with real-world statistics. For example, we classify bias as under-representational when “the plumber” is translated as masculine, reflecting real data that plumbers are rarely women (the percentage of male “Plumbers and heating and ventilating engineers” in the UK was equal to 98% in 2023). Gender bias is considered stereotypical when the gender assignment aligns with a negative generalisation, such as translating “the professor” as masculine, even though professors are equally often women (the percentage of male “Higher education teaching professionals” in the UK is equal to 56% in 2023). Finally, bias is categorised as algorithmic when the gender assignment likely results from a machine translation algorithmic error, rather than being based on the low representation of a specific gender in linguistic corpora. Thus, the KG developed in this study serves as a valuable resource for examining gender bias in machine translation systems and offers critical insights into gender representation across various professional sectors. This comprehensive framework enables a detailed understanding of how occupations are “gendered” both in the actual labour market and within the textual data used to train and test machine learning systems.

## Results

### Gender occupational segregation results based on raw data drawn from the EU-LFS

**Fig. 3:**
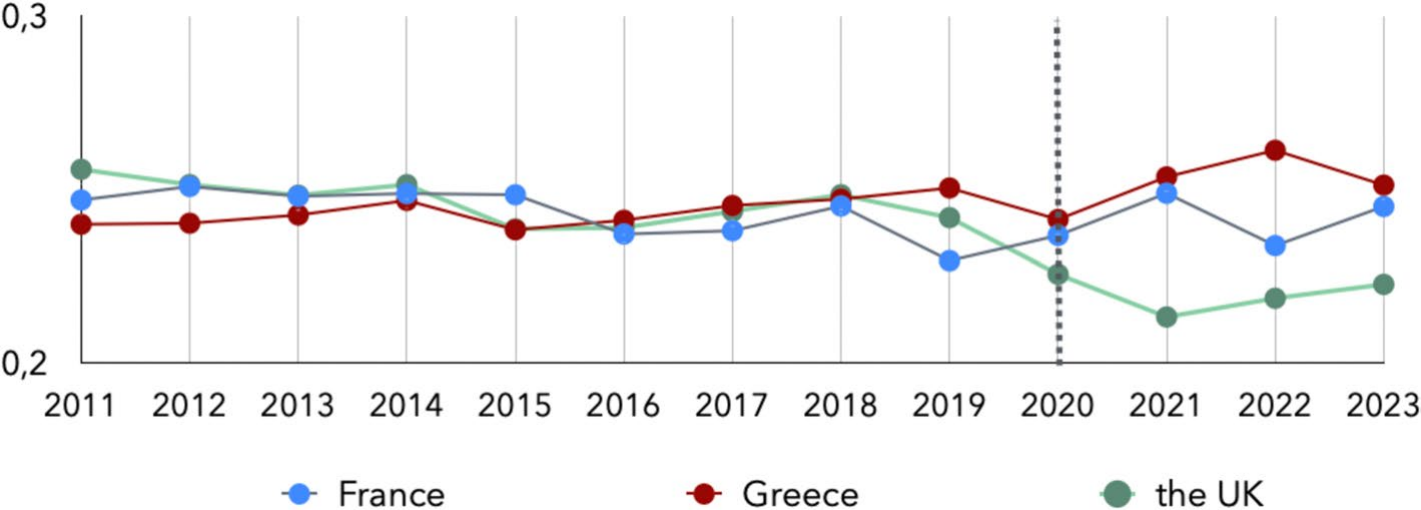
Dissimilarity index France, Greece and the UK, 2011–2023

The Duncan dissimilarity index (DI), tracking how evenly men and women are distributed across occupations, was calculated for the three countries from 2011 to 2023 (Fig. 3) based on the gendered distributions using raw data extracted from the EU-LFS datasets and the occupations at ISCO-08 3-digit level. As earlier noted, the dissimilarity index for the UK for the years 2020–2023 are estimated using the Official Statistics and the SOC 2020 classification.

Between 2011 and 2023, the DI reveals differing trends in occupational gender segregation across France, Greece, and the UK. In Greece, the DI rose steadily from 0.240 in 2011 to a peak of 0.261 in 2022, indicating a gradual increase in segregation. France exhibits a similar overall range but with more pronounced year-to-year fluctuations; for instance, the index decreases in 2016 (0.237), 2019 (0.230), and 2022 (0.234), though these shifts do not indicate a consistent long-term trend. In contrast, the UK shows a decline in the DI from 0.256 in 2011 to 0.242 in 2019, suggesting a modest improvement in gender balance. From 2020 onward, the DI continues to decline, however these values are based on official UK statistics using the updated SOC 2020 occupational classification, which may affect comparability with earlier years.

The statistical analysis outcomes of the descriptive analysis performed using raw data drawn from the EU-LFS for Greece and France and the Official Statistics Office for the UK are now discussed. In the UK for the year 2023, the data shows a strong concentration of men in technical, engineering, and skilled trades. Occupations such as aircraft maintenance, vehicle trades, and electrical engineering are overwhelmingly male with a percentage almost equal to 99%. Similarly, roles in construction, such as production managers and civil engineers remain heavily male-dominated (with percentages almost equal to 91% and 92% respectively). A wide range of manual and technical jobs, including electrical service mechanics, stonemasons, and rail maintenance operatives, also show little to no female representation, with some occupations like electrical service and maintenance roles being 100% male. On the other hand, women are highly represented in healthcare, education, and administrative roles, with some jobs having over 95% female workers, for example, nursery teachers, child-minders, secretaries, and florists. A few roles, such as midwives and nannies, are entirely female. In contrast, professions like doctors, lawyers, and data entry roles show a more balanced gender split, ranging from roughly 44% to 55% female representation. A detailed description of the male-dominated, female-dominated and balanced occupations are exhibited in Figs. 7 and 8 in the Appendix.

Similarly, the analysis reveals a significant number of occupations with 100% male employment in Greece, particularly in technical, mechanical, and transport-related fields. These include occupations such as ICT service managers, mining supervisors, locomotive engine drivers, and mobile plant operators. Even beyond these, many other jobs, such as heavy truck drivers, building finishers, and electrical equipment installers, have male representation exceeding 95%. This concentration reflects a persistent pattern of occupational gender segregation, with men dominating roles that involve physical labour, machinery, and infrastructure. The analysis also shows that women remain highly concentrated in caregiving, clerical, and service roles, such as child-care, nursing, and cleaning. For example, occupations like secretaries, librarians, and hairdressers continue to be predominantly female. A few fields are closer to gender balance, including finance professionals, architects and planners, and social and religious workers. Figure 9 in the Appendix highlights male-dominated occupations in Greece for the year 2023 and Fig. 10 the female-dominated and gender-balanced occupations.

The data analysis for France for the year 2023 shows a strong concentration of men in skilled trades, transport, and technical fields. Several roles, including forestry workers, are entirely male, while many others, such as building finishers, mining labourers, and electrical equipment installers, have male representation exceeding 95%. Additional occupations like mobile plant operators, mechanics, and welders also show clear male dominance. ICT service managers and construction supervisors, though slightly lower, still report over 90% male employment. Compared to Greece, the pattern is broadly similar: men dominate many of the same sectors in both countries, especially in manual and infrastructure-related roles. However, there is only one occupation with exactly 100% male representation, suggesting marginally more female presence in highly male-skewed fields. The data also reveals that women are predominantly employed in caregiving, administrative, and personal service roles. For instance, approximately 96% of childcare workers and teachers’ aides, 95% of general secretaries, and 92% of personal care workers in health services are women. Similarly high female representation is seen among hairdressers (88.10%) and nursing and midwifery professionals (87.25%). Some professions, however, are evenly split by gender, such as vocational and higher education teachers (50/50). Other roles, like medical doctors and sales agents, show near-gender balance, with women comprising 53% and 49% respectively. Figure 11 depicts male-dominated occupations in France for 2023 and Fig. 12 the female-dominated and gender-balanced ones.

In conclusion, the analysis of gender distribution in occupations across Greece, France, and the UK reveals consistent patterns of gender disparity. Technical and manual trades are predominantly male across all three countries, indicating a strong gender imbalance in sectors requiring technical skills and manual labour. Care-giving and administrative roles are predominantly female, reflecting societal trends where women are more represented in these occupations. Professional and managerial roles, while still showing gender imbalances, are closer to achieving gender parity compared to technical and manual trades.

**Fig. 4:**
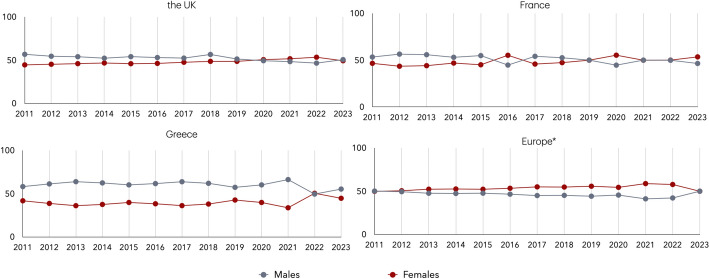
Gender distribution of medical doctors (ISCO-08 code 221) in the UK, France, Greece, and the average across 28 European countries (2011–2023). *Note*: *28 European countries: AT, BE, HR, CY, CH, CZ, DE, DK, EE, ES, FI, FR, GR, HU, IE, IS, IT, LV, LT, LU, NO, NL, PT, PL, RO, SE, SK, UK. For the UK, the figures from 2020 to 2023 are based on official statistics using the updated SOC 2020 classification for Medical Practitioners, which may affect direct comparability with earlier years

In the context of automated translation systems, a commonly used example of gender bias is the translation of the sentence “The doctor gave the forceps to the nurse” by Google Translate, which is rendered in Greek as “Ο γιατρός έδωσε τη λαβίδα στη νοσοκόμα”. The same applies also for French, i.e. the translation becomes “Le médecin a donné les forceps à l’infirmière”. Thus, the automated translation systems assign a male gender to the doctor and a female gender to the nurse, reinforcing gender stereotypic roles. ChatGPT and DeepL also provide the same translation. However, shifts in gender distribution among doctors over the past decade challenge the perception of the medical profession as being predominantly male. To provide more evidence for this shift, we estimate the gender composition of medical doctors (ISCO-08 code 221) in the UK, France, Greece, and 25 additional European countries and depict the evolution of gender representation in the medical profession both within these three countries and across Europe using raw data drawn from the EU-LFS (Fig. 4).

**Fig. 5:**
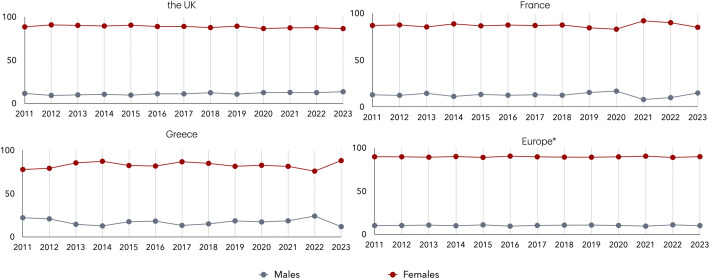
Gender distribution of nursing and midwifery professionals (ISCO-08 code 222) in the UK, France, Greece, and the average across 28 European countries (2011–2023). *Note*: *28 European countries: AT, BE, HR, CY, CH, CZ, DE, DK, EE, ES, FI, FR, GR, HU, IE, IS, IT, LV, LT, LU, NO, NL, PT, PL, RO, SE, SK, UK. For the UK, the data from 2020 to 2023 are based on official statistics using the SOC 2020 classification for nursing professionals, which may affect comparability with earlier years

Between 2011 and 2023, the gender distribution among medical doctors in the studied countries, and the broader European context shows a general trend toward gender balance, with several countries reaching or surpassing parity in recent years. In the UK, the proportion of female medical doctors increased steadily, surpassing 50% in 2020 and peaking at 53.4% in 2022, before slightly declining to 49.3% in 2023. In France, female representation also rose over time, reaching 53.5% in 2023 after fluctuating around parity in previous years. Greece exhibited greater year-to-year variation, but the proportion of female doctors has shown an upward trend overall, peaking at 50.5% in 2022. Across Europe, the share of female medical doctors has consistently increased, exceeding male representation from 2013 onwards and reaching 58.8% in 2021. This trend calls into question the automatic interpretation or translation of “the doctor” as male, framing such usage as a form of *stereotypical bias*. Moreover, in the examined period, the gender distribution of nursing and midwifery professionals (ISCO-08 code 222) in the UK, France, Greece, and across Europe consistently shows a strong female dominance (Fig. 5).

While there are slight variations over time and across countries, women overwhelmingly dominate the profession in all cases. This persistent pattern highlights the gendered nature of nursing and reinforces the common tendency to translate “the nurse” as female. Such linguistic conventions reflect an *under-representational bias*, as they obscure the presence of men in the profession and contribute to the reinforcement of occupational gender stereotypical roles.

**Fig. 6:**
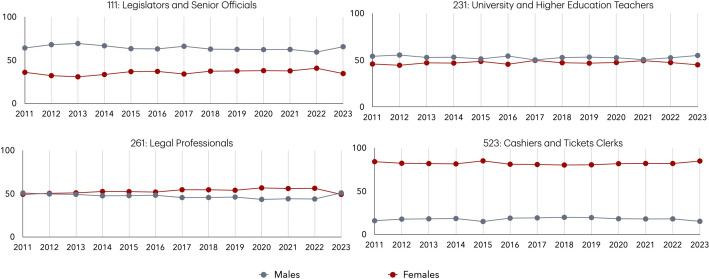
Gender distribution of legislators and senior officials (ISCO-08 code 111), university and higher education teachers (ISCO-08 code 231), legal professionals (ISCO-08 code 261), and cashier and ticket clerks (ISCO-08 code 523) across 28 European countries (2011–2023). *Note*: *28 European countries: AT, BE, HR, CY, CH, CZ, DE, DK, EE, ES, FI, FR, GR, HU, IE, IS, IT, LV, LT, LU, NO, NL, PT, PL, RO, SE, SK, UK

**Fig. 7:**
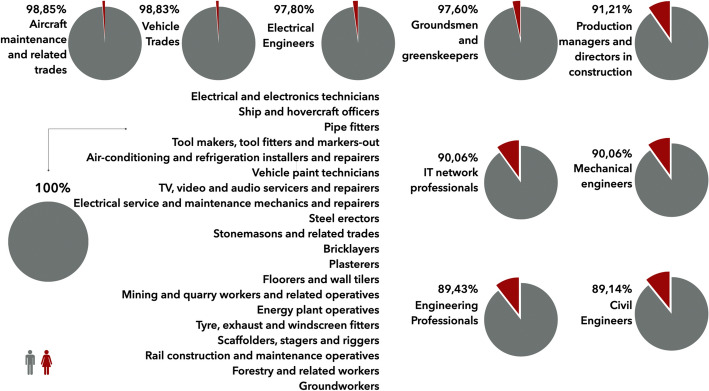
“Male” occupations, UK, 2023

**Fig. 8:**
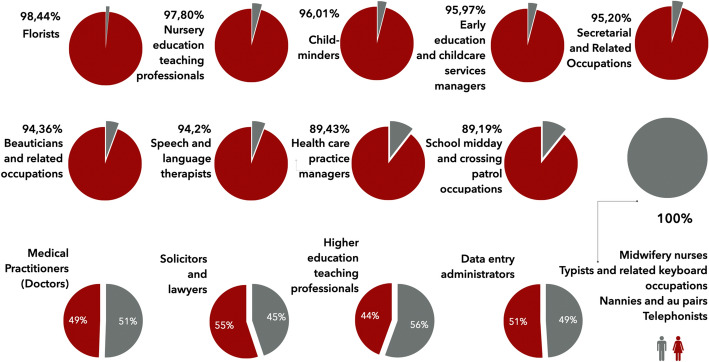
“Female” and “gender-balanced” occupations, UK, 2023

**Fig. 9:**
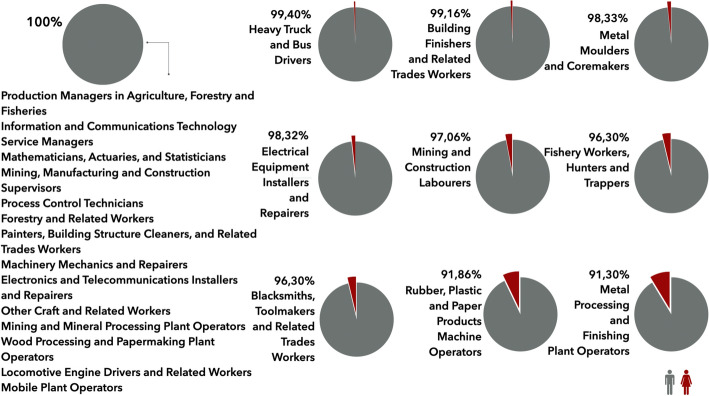
“Male” occupations, Greece, 2023

**Fig. 10:**
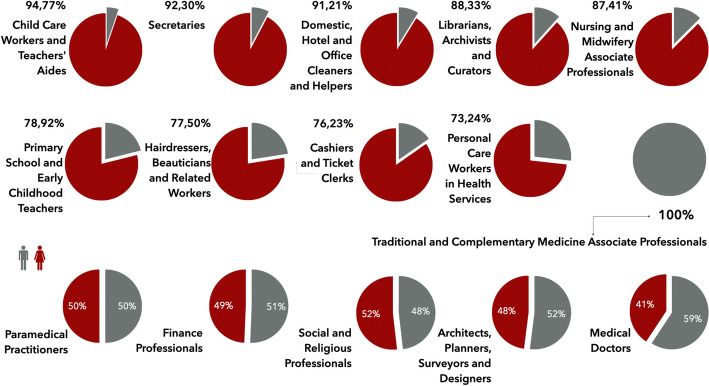
“Female” and “gender-balanced” occupations, Greece, 2023

**Fig. 11:**
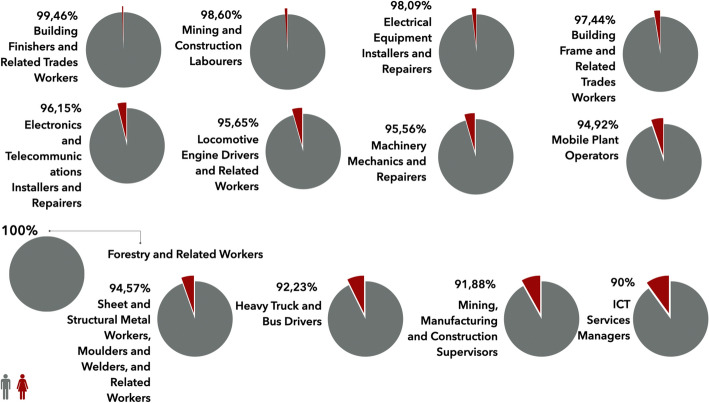
“Male” occupations, France, 2023

**Fig. 12:**
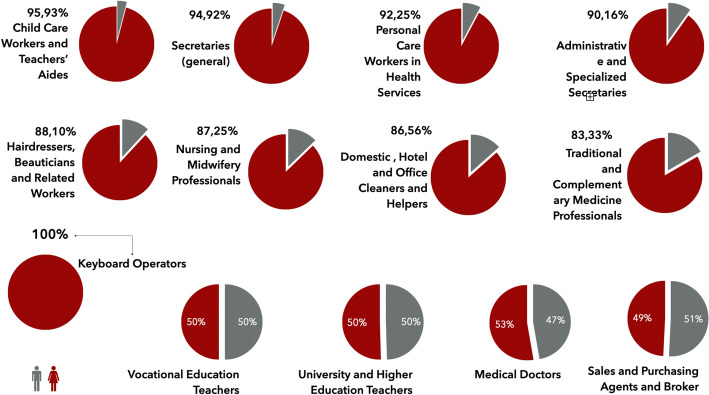
“Female” and “gender-balanced” occupations, France, 2023

Other examples involving the machine translation of sentences that include an occupation such as *manager*, *legislator, lawyer*, *nursery teacher*, *cashier clerk*, *University professor*, *engineer*, *secretary*, and *cleaner* reveal a consistent pattern of gendered assumptions both in Google translate, ChatGPT and DeepL: manager, legislator, lawyer, University professor, and engineer are translated into French and Greek as being male, while the positions of nursery teacher, cashier clerk, secretary, and cleaner are translated as being female. However, our analysis at a European level (Fig. 6) shows that these translation outputs do not reliably reflect the actual gender distribution in these occupations. For example, legal professionals (ISCO-08 code 261) have consistently had a majority of female workers, yet the term *lawyer* is routinely translated as male. Similarly, University professors (ISCO-08 code 231) show a near gender balance in most years, and women represent over 40% of legislators (ISCO-08 code 111), yet both professions are still predominantly associated with male translations. Even in roles such as cleaners, where men make up around 20%, the translation defaults to female. These patterns indicate that machine translation systems are not simply mirroring labour market data but are instead reproducing and reinforcing occupational gender stereotypes, often in contradiction to the actual distribution of women and men in these roles.

### Textual data analysis and comparison with official labour statistics

In this study we conducted experiments in three languages: English, French, and Greek, using the WMT training set (Bojar et al. 2014) and the C4 dataset (Raffel et al. 2020). The WMT training set, developed for the Workshop on Machine Translation (WMT), are widely used benchmark datasets for training and evaluating machine translation systems. These corpora consist of sentence-aligned bilingual texts, allowing for supervised learning of translation models. On the other hand, C4 is a large-scale dataset developed through an extensive cleaning process to remove repetitive, non-informative, or generic content, and toxic or inappropriate material. The result is a high-quality corpus of texts, designed to support large-scale natural language processing tasks. These datasets were chosen due to C4’s widespread use in pretraining large language models (LLMs) and WMT’s prominence in training machine translation (MT) systems, including LLMs adapted for MT tasks. Our main emphasis was on the WMT training set, given its specific role in MT system training, which may introduce certain biases. Table 1 presents the number of instances analysed from both the WMT and C4 datasets for each language: English, French, and Greek. While both datasets provided substantial data for English and French, only C4 contained instances for Greek, as the WMT dataset does not include a Greek subset.

**Table 1:** Number of instances analysed from WMT and C4 datasets for English, French, and Greek

Text instances analysed	English	French	Greek	Total
WMT	77,079	28,730	N/A	105,809
C4	54,298	14,638	26,175	95,111
Total	131,377	43,368	26,175	200,920

It is important to note that a total of 28,289 occupational terms were detected in the analysed texts, of which gender could be identified in 4,248 cases. Table 2 presents the distribution of gender-identifiable and unidentified occupational terms across English, French, and Greek texts. French and Greek exhibit a high rate of gender identification, with the majority of terms marked as masculine (1,857 and 306 respectively), while feminine forms are less frequent. In contrast, English shows a far lower rate of identifiable gender. Only 1,315 of 23,831 terms (5.5%) were identified as either masculine or feminine, reflecting the largely gender-neutral structure of the English language, where grammatical gender is not typically marked. As a result, over 94% of occupational terms in English texts were classified as “unidentified”. The large volume of unidentified terms in English poses challenges for cross-linguistic comparisons and underscores the need for caution when translating from English into grammatically gendered languages.

**Table 2:** Gender identification of occupational terms in English, French, and Greek texts

Gender identification	English texts	French texts	Greek texts	Total
Masculine	849	1,857	306	3,012
Feminine	466	733	37	1,236
Total	1,315	2,590	343	4,248
Unidentified	22,516	1,410	115	24,041
Total	23,831	4,000	458	28,289

Table 3 presents the male and female counts from official labour data alongside gender-identifiable terms found in the 131,377 analysed text for each ISCO-08 major occupational group for the UK. It includes male-to-female (M/F) ratios for both sources, odds ratios indicating the degree of over- or under-representation of men in text, and p-values from Fisher’s exact tests assessing statistical significance. Statistically significant disparities suggest systematic gender bias in the way occupations are referenced in text compared to actual labour market composition. Thus, the comparison between official employment statistics and textual representations reveals significant disparities in how occupational gender is portrayed. In several high-profile categories such as Managers and Professionals, men are overrepresented in text compared to their actual share in the labour market. This is also particularly evident among Technicians and Associate Professionals and Clerical Support Workers, where the odds ratios are 4.32 and 4.03 respectively (both at *p* < 0.001), indicating that male terms appear in text at disproportionately higher rates despite official data showing gender parity or female dominance. Conversely, for Service and Sales Workers, where women significantly outnumber men in the workforce (M/F ratio: 0.49), text shows a smaller gender gap (M/F ratio: 0.81), resulting in a notably low odds ratio of 0.10 (*p* < 0.001), suggesting underrepresentation of women in this category in textual references. Other male-dominated occupations such as Craft and Related Trades Workers and Plant and Machine Operators show the opposite bias, with text suggesting less male dominance than the data supports, possibly reflecting more balanced portrayals in these cases. However, even here, significant differences are observed (*p* < 0.001). Elementary Occupations and Armed Forces show no statistically significant discrepancies between text and official data, however a smaller number of gender-identifiable references in those categories is detected.

**Table 3:** Comparison of gender representation in occupational terms across ISCO-08 major groups: official labour force statistics vs. textual references for the UK

ISCO	Major groups	Official N (M/F)	Text N (M/F)	M/F ratio official	M/F ratio text	Odds ratio	*p*-value
1	Managers, directors and senior official	2,418,300/1,412,400	136/35	1.71	3.89	2.25	< .001***
2	Professionals	4,395,700/4,201,500	164/108	1,05	1.52	1.45	.003**
3	Technicians and associate professionals	1,934,800/2,091,500	318/72	0.93	4.42	4.32	< .001***
4	Clerical support workers	926,000/2,127,700	65/42	0.44	1.55	4.03	< .001***
5	Service and sales workers	1,892,700/3,871,200	141/175	0.49	0.81	0.10	< .001***
6	Skilled agricultural, forestry and fishery workers	321,600/35,800	4/0	8.98			
7	Craft and related trades workers	2,484,600/96,000	8/4	25.88	2.00	0.08	< .001***
8	Plant and machine operators, and assemblers	1,391,600/192,700	8/23	7.22	0.35	0.05	< .001***
9	Elementary occupations	1,429,300/1,233,900	4/6	1.16	0.67	0.60	.531
0	Armed forces	74,100/10,200	1/1	7.27	1	0.14	.299

*** *p* < 0.001, ** *p* < 0.01 (Fisher’s exact test for gender distribution differences between official statistics and in-text portrayal)

Table 4 presents male and female counts from official labour force statistics alongside gender-identifiable terms extracted from the 43,368 analysed texts for each ISCO-08 major occupational group. Overall, the comparison reveals discrepancies in occupational gender portrayal. In the Managers category the difference is not statistically significant (*p* = 0.129). In contrast, Professionals show a significant textual skew toward men despite near parity in employment (*p* = 0.001). More striking are the divergences observed in mid-level occupational groups. Technicians and Associate Professionals, where women slightly outnumber men in the workforce, appear with an M/F ratio of 1.43 in text, yielding an odds ratio of 1.91 (*p* < 0.001), clearly favouring male-coded references. Similarly, Clerical Support Workers, who are predominantly women in the labour force, are referenced disproportionately as men in text (OR = 10.88, *p* < 0.001). These differences indicate strong textual masculinisation of female-majority professions. Conversely, for Service and Sales Workers, the M/F ratio is closer between text and official data but still shows a significant underrepresentation of women (OR = 2.10, *p* < 0.001). A similar pattern is found in Skilled Agricultural, Forestry and Fishery Workers, where the already male-dominated profession (M/F = 3.47) is further exaggerated in text (M/F = 13.8, OR = 5.23, *p* < 0.001). Interestingly, in male-dominated fields like Craft and Related Trades Workers and Plant and Machine Operators, the text downplays male dominance. For example, Craft workers have an M/F ratio of 9.09 in the official data but near parity in text (M/F = 1.02), reflected in a low odds ratio of 0.11 (*p* < 0.001). Finally, Elementary Occupations show the most extreme distortion, with women forming the majority in official data but men being overwhelmingly referenced in text (OR = 49.98, *p* < 0.001). In contrast, Armed Forces, while heavily male in both sources, do not show a statistically significant difference (*p* = 0.447), likely due to the small number of gender-identifiable references.

**Table 4:** Comparison of gender representation in occupational terms across ISCO-08 major groups: official labour force statistics vs. textual references for France

ISCO	Major groups	Official N (M/F)	Text N (M/F)	M/F ratio official	M/F ratio text	Odds ratio	*p*-value
1	Managers	2,028,100/806,400	94/49	2.52	1.92	1.31	.129
2	Professionals	3,443,900/3,520,500	185/121	0.98	1.53	1.56	.001**
3	Technicians and associate professionals	2,184,100/2,909,600	213/149	0.75	1.43	1.91	< .001***
4	Clerical support workers	645,100/1,640,500	30/7	0.39	4.29	10.88	< .001***
5	Service and sales workers	1,313,600/2,730,800	117/120	0.48	0.98	2.10	< .001***
6	Skilled agricultural, forestry and fishery workers	524,100/151,100	138/10	3.47	13.80	5.23	< .001***
7	Craft and related trades workers	2,426,100/266,900	227/223	9.09	1.02	0.11	< .001***
8	Plant and machine operators, and assemblers	1,456,700/309,500	88/33	4.71	2.67	0.56	< .001***
9	Elementary occupations	974,900/1,371,400	759/19	0.71	39.95	49.98	< .001***
0	Armed forces	156,900/22,400	6/2	7.01	3.00	0.43	0.447

*** *p* < 0.001, ** *p* < 0.01 (Fisher’s exact test for gender distribution differences between official statistics and in-text portrayal)

Table 5 presents the differences between official labour statistics and gender detection in approximately 26,000 Greek texts. Among Professionals, where official employment data shows near gender parity (M/F ratio = 0.82), textual references are overwhelmingly male-coded (M/F ratio = 10.11), with an odds ratio of 0.08 (*p* < 0.001), indicating a strong and significant overrepresentation of men. This pattern is even more pronounced among Technicians and Associate Professionals, where the official male majority is modest (M/F ratio = 1.44), yet textual references skew dramatically male (M/F ratio = 25.4), producing an odds ratio of 0.06 (*p* < 0.001). Similarly, Service and Sales Workers, a nearly gender-balanced group in the workforce (M/F ratio = 0.91), are heavily masculinised in text (M/F ratio = 5.67), with an odds ratio of 0.16 (*p* < 0.001).

**Table 5:** Comparison of gender representation in occupational terms across ISCO-08 major groups: official labour force statistics vs. textual references for Greece

ISCO-08 code	Major groups	Official N (M/F)	Text N (M/F)	M/F ratio official	M/F ratio text	Odds ratio	*p*-value
1	Managers	117,500/40,900	27/13	2.87	2.08	1.38	.370
2	Professionals	415,400/506,600	91/9	0.82	10.11	0.08	< .001***
3	Technicians and associate professionals	161,200/111,700	127/5	1.44	25.4	0.06	< .001***
4	Clerical support workers	183,500/277,400	3/1	0.66	3.0	0.22	.310
5	Service and sales workers	455,200/499,500	34/6	0.91	5.67	0.16	< .001***
6	Skilled agricultural, forestry and fishery workers	243,400/145,700	0/0	1.67			
7	Craft and related trades workers	373,900/30,700	0/0	12.18			
8	Plant and machine operators, and assemblers	261,100/31,300	5/0	8.34			
9	Elementary occupations	123,000/141,400	0/0	0.87			
0	Armed forces	57,600/9,200	19/3	6.26	6.33	0.99	1.000

*** *p* < 0.001 (Fisher’s Exact Test for gender distribution differences between official statistics and in-text portrayal)

## Limitations

This study is subject to certain limitations related to the structure of the data sources, the alignment of occupational classification systems over time, and the methodology of gender identification in text. First, the statistics concerning labour market data used in our analysis are drawn from the EU-LFS survey, where respondents’ occupations are provided at the 3-digit level of the ISCO-08 classification. The more granular 4-digit level, which could provide finer occupational detail and improve precision in mapping gender representation, is not made publicly available by EUROSTAT. However, the available data still provide a valid overview of gender representation across occupations. Furthermore, occupational classifications differ across countries and over time. In particular, for the United Kingdom, official statistics from 2020 onward employ the SOC 2020 classification, which does not align directly with the ISCO-08 classification. We note here that there is no direct mapping between ISCO-08 and SOC 2020 at all levels except the 4-digit level. To ensure comparability between official labour statistics and the textual data for the UK, we use data from 2019, which adheres to the ISCO-08 occupational classification system. Moreover, in the analysis of the dissimilarity index and the longitudinal comparisons presented in Figs. 3, 4 and 5, it should be noted that UK data from 2020 to 2023 are based on the SOC 2020 classification. Therefore, the gender distribution in Fig. 4 for the UK is based on the percentages of Medical Doctors (ISCO-08 code 221) for the years 2011–2019 and Medical Practitioners (SOC 2020 code 221) for the years 2020–2023. Similarly, in Fig. 5 Nursing and Midwifery Professionals (ISCO-08 code 222) is used for the years 2011–2019 and Nursing Professionals (SOC 2020 code 223) for 2020–2023. Thus, during the last four years this divergence in the classification may affect comparability with earlier ISCO-08-based data. Additionally, the official labour statistics results used in this study also carry inherent limitations. These figures are derived from survey-based estimates rather than exhaustive census data. While the EU-LFS is designed to produce reliable results, all such data are subject to sampling variability. Consequently, short-term fluctuations in occupational gender distributions should be interpreted with caution.

In relation to the textual analysis, a substantial proportion of occupational mentions extracted from the textual corpus, particularly in English, are unidentified in relation to their gender. This is potentially due to the grammatical structure of the English language, where occupational terms are typically ungendered. In our dataset, only 4,248 of 28,289 textual occupation mentions were associated with an identifiable gender, with most unidentified instances (22,516) occurring in English texts. Thus, although occupations were detected in 28,289 instances across the corpus, gender was identifiable in only 15% of cases. Detection rates varied significantly by language: gender was identified in just 5.5% of English texts, compared to 64.8% in French and 74.9% in Greek. The limited number of gender-identified occupational terms in the textual data constrains the extent to which statistical comparisons can be made with official labour statistics beyond the ISCO-08 major occupational groups. This limitation not only affects the comparability between official statistics and text analysis results but also highlights the need for caution when translating from English into grammatically gendered languages. Furthermore, we acknowledge that the Greek dataset includes a relatively small number of gendered occupational references.

## Conclusions and future aspects

In this paper, we investigated the interconnected dynamics between gender stereotypes, labour market gender inequalities, and gender bias in machine learning systems, with a particular focus on text-based occupational representations and automated translation technologies. This approach allows for a more nuanced understanding of how biases are embedded not only in algorithmic processes but also in the datasets themselves, which often reflects historical, societal, and linguistic structures. Our analysis proves that the datasets, specifically WMT that is used to train and evaluate automated translation systems, often encode existing gender biases, which are then reproduced in the system outputs, particularly in relation to occupational terms. By decoupling these factors, we aim to offer clearer insights into the root causes of bias and highlight the importance of addressing not only algorithmic adjustments but also the underlying data sources used in training and evaluation of ML models. This study underscores the significant challenges posed by gender bias in machine learning and machine translation systems, particularly in the representation of occupational roles. The development of the Knowledge Graph introduces an innovative approach to integrating real-world labour statistics with the textual corpora used in MT training. By consolidating real world statistics into a single Knowledge Graph, the work presented in this paper enables the identification and analysis of discrepancies between gender distributions in the actual labour market and those present in machine learning training datasets.

The comparative analysis of gender representation across official labour force statistics and textual data reveals consistent and statistically significant disparities, indicating systematic masculinisation of several occupations in written language. Evidence shows that, across the UK, France, and Greece, male-coded references frequently exceed what would be expected based on actual employment figures. In high-prestige occupations such as Managers and Professionals, men are often overrepresented in text despite more balanced or even female-majority workforce participation. This pattern is even more pronounced in mid-level occupations such as Technicians and Associate Professionals and Clerical Support Workers, where odds ratios consistently indicate significant male overrepresentation in text despite gender parity or female dominance in the labour force. These findings demonstrate that gender bias in occupational portrayal is pervasive and structured, with implications for how automated systems, such as machine translation tools and language models, reproduce and potentially reinforce gender stereotypes.

The misalignment between textual analysis and official statistics should not be seen as an indication that the data does not reflect reality, nor that the methodology used to detect gender in texts is inherently flawed. Instead, it underscores the importance of acknowledging the biases embedded in the datasets. The divergences observed point to broader societal and contextual biases that shape the way gender is represented across different data sources, reinforcing the importance of critically examining these factors when interpreting results. The observed gender imbalances between textual corpora and real-world statistics are a reflection of deeper, systemic biases inherent in the data sources themselves. These biases often stem from various factors, including historical, societal, and methodological influences that shape how data is collected, interpreted, and used in machine learning models. While factors in our methodology such as sampling bias, linguistic nuances, and technical challenges could play a role in the discrepancies, these issues alone do not fully explain the extent of the differences. Data bias, and specifically gender bias in data, is a well-documented issue (Barocas et al. 2023; Mehrabi et al. 2021; Binns 2018; Leavy et al. 2020), with multiple forms contributing to the discrepancies we observe, such as reporting bias, where certain events or behaviours are over- or under-reported, historical bias, which arises from using data that reflects outdated societal norms and structures, and group attribution bias, where certain traits or characteristics are unfairly generalised. Although some alignment between real-world occupational imbalances and textual representations can be attributed to actual gender disparities in certain fields -such as male-dominated sectors- this alignment is only part of the story. The datasets, whether drawn from historical sources or curated by certain institutions, may reflect the perspectives and priorities of historically dominant social groups. These demographic imbalances in data curation and publication can result in skewed representations of gender within professional fields. Moreover, the static nature of many datasets means that they may not accurately capture the ongoing evolution of gender dynamics in the labour market, thereby exacerbating discrepancies between contemporary gender roles and those detected from historical or sector-specific textual sources.

Future research will focus on broadening the proposed methodology to encompass a wider range of datasets, thereby enhancing the statistical analysis available for commonly used training corpora in large language models. Ultimately, the Knowledge Graph could be utilised to pinpoint the sources of misalignments, whether they stem from the datasets, inherent algorithmic biases, or a combination of both. An additional future step involves incorporating new and emerging occupations as they become available by the Technical Working Group responsible for updating the ISCO-08 classification.

## Data Availability

The data supporting Table 6 are available at the ILOSTAT database: https://ilostat.ilo.org/resources/concepts-and-definitions/classification-occupation/. The graphs presented in Figs. 7–12 and Figs. 3–6 are based on our analysis of anonymised raw microdata provided by the EUROSTAT Microdata Access Team for scientific research purposes. Additionally, publicly available data from NOMIS -the UK’s official source for census and labour market statistics- have been used in the construction of Fig. 3 (United Kingdom, years 2020–2023), accessible at: https://www.nomisweb.co.uk/datasets/aps218/reports/employment-by-occupation?compare=K02000001. Furthermore, the experiments reported in this study utilised the WMT training dataset, available at https://huggingface.co/datasets/wmt/wmt14, and the C4 dataset, available at https://huggingface.co/datasets/allenai/c4?row=1.

## Appendix: Occupational classifications, technical specifications, and additional results

### A1. The ISCO-08 classification

The ISCO-08 major groups are the following:

- **0-Armed Forces Occupations** Armed forces occupations include all jobs held by members of the armed forces. Members of the armed forces are those personnel who are currently serving in the armed forces, including auxiliary services, whether on a voluntary or compulsory basis, and who are not free to accept civilian employment and are subject to military discipline. Included are regular members of the army, navy, air force and other military services, as well as conscripts enrolled for military training or other service for a specified period.
- **1-Managers** Managers plan, direct, coordinate and evaluate the overall activities of enterprises, governments and other organisations, or of organisational units within them, and formulate and review their policies, laws, rules and regulations. Competent performance in most occupations in this major group requires skills at the fourth ISCO skill level, except for Sub-major group 14: Hospitality, Retail and Other Services Managers, for which skills at the third ISCO skill level are generally required.
- **2-Professionals** Professionals increase the existing stock of knowledge; apply scientific or artistic concepts and theories; teach about the foregoing in a systematic manner; or engage in any combination of these activities. Competent performance in most occupations in this major group requires skills at the fourth ISCO skill level.
- **3-Technicians and Associate Professionals** Technicians and associate professionals perform technical and related tasks connected with research and the application of scientific or artistic concepts and operational methods, and government or business regulations. Competent performance in most occupations in this major group requires skills at the third ISCO skill level.
- **4-Clerical Support Workers** Clerical support workers record, organise, store, compute and retrieve information, and perform a number of clerical duties in connection with money-handling operations, travel arrangements, requests for information, and appointments. Competent performance in most occupations in this major group requires skills at the second ISCO skill level.
- **5-Service and Sales Workers** Service and sales workers provide personal and protective services related to travel, housekeeping, catering, personal care, or protection against fire and unlawful acts, or demonstrate and sell goods in wholesale or retail shops and similar establishments, as well as at stalls and on markets. Competent performance in most occupations in this major group requires skills at the second ISCO skill level.
- **6-Skilled Agricultural, Forestry and Fishery Workers** Skilled agricultural, forestry and fishery workers grow and harvest field or tree and shrub crops, gather wild fruits and plants, breed, tend or hunt animals, produce a variety of animal husbandry products; cultivate, conserve and exploit forests; breed or catch fish; and cultivate or gather other forms of aquatic life in order to provide food, shelter and income for themselves and their households. Competent performance in most occupations in this major group requires skills at the second ISCO skill level.
- **7-Craft and Related Trades Workers** Craft and related trades workers apply specific technical and practical knowledge and skills in the fields to construct and maintain buildings; form metal; erect metal structures; set machine tools or make, fit, maintain and repair machinery, equipment or tools; carry out printing work; and produce or process foodstuffs, textiles and wooden, metal and other articles, including handicraft goods. Competent performance in most occupations in this major group requires skills at the second ISCO skill level. The work is carried out by hand and by hand-powered and other tools which are used to reduce the amount of physical effort and time required for specific tasks, as well as to improve the quality of the products. The tasks call for an understanding of all stages of the production process, the materials and tools used, and the nature and purpose of the final product.
- **8-Plant and Machine Operators, and Assemblers** Plant and machine operators, and assemblers operate and monitor industrial and agricultural machinery and equipment on the spot or by remote control; drive and operate trains, motor vehicles and mobile machinery and equipment; or assemble products from component parts according to strict specifications and procedures. Competent performance in most occupations in this major group requires skills at the second ISCO skill level. The work mainly calls for experience with and an understanding of industrial and agricultural machinery and equipment as well as an ability to cope with machine-paced operations and to adapt to technological innovations.
- **9-Elementary Occupations** Elementary occupations involve the performance of simple and routine tasks which may require the use of hand-held tools and considerable physical effort. Most occupations in this major group require skills at the first ISCO skill level.

Table 6 presents the number of subgroups, minor groups and unit groups for the ten major groups.

**Table 6:** Number of groups at each level, ISCO-08. *Source**:* ILO (2023b: p. 22)

Major groups	Subgroups	Minor groups	Unit groups
1: Managers	4	11	31
2: Professionals	6	27	92
3: Technicians and associate professionals	5	20	84
4: Clerical support workers	4	8	29
5: Service and sales workers	4	13	40
6: Skilled agricultural, forestry and fishery workers	3	9	18
7: Craft and related trades workers	5	14	66
8: Plant and machine operators, and assemblers	3	14	40
9: Elementary occupations	6	11	33
0: Armed forces occupations	3	3	3
Total	43	130	436

ISCO-08 is presently undergoing a revision process conducted by a Technical Working Group (TWG) and the ILO’s statistics department (ILO 2023b). The aims of this revision include keeping ISCO current with developments in the world of work by refining and enhancing the ISCO skill model and structure, and to modernise ISCO by e.g. including new emerging jobs. In anticipation of the forthcoming revision of ISCO-08 before 2030, enabling countries to adopt the new version for their census operations, the TWG was convened in 2021 to aid the ILO, comprising representatives from 36 countries. Numerous suggestions and modifications were proposed and currently being considered, such as updating the ISCO structure, incorporating new and emerging occupations, the creation of job families or job clusters, the improvement of the conceptual clarity for various categories and identification of systematic treatment for these, among others. Given the swift pace of technological progress and the wealth of available data sources, it became imperative to investigate novel approaches for ensuring the prevalence of ISCO-08. Thus, in 2022, a proof of concept (POC) was initiated aiming at employing AI and emerging data sources for potential use in future ISCO-08 updates. It utilised Machine Learning (ML) and Natural Language Processing (NLP) methodologies to parse the extensive dataset, rendering it amenable for analysis. The POC developed a composite index for New and Emerging Occupations, assessing the probability of a job title qualifying as a potential new and emerging occupation. To validate the existence of such candidates across different countries/regions globally, the job titles underwent cross-referencing with ISCO-08 and a range of recently revised National and Regional Occupational Classifications via semantic textual similarity. Any candidates identified for the ISCO-08 revision will undergo validation by classification experts. Readers can consult the European Commission (2021) report and ILO (2023b) to inform themselves in relation to the feasibility study on identifying new and emerging occupations candidates using data from online job advertisements to support the revision of ISCO-08.

### A2. The standard occupational classification (SOC 2020)

In the UK, up until 2019, the ISCO-08 classification was employed as a requirement to align with the taxonomy used in the EU-LFS, while concurrently, the UK has maintained the use of the Standard Occupational Classification for the UK. The Standard Occupational Classification (SOC), first introduced in 1990 and maintained by the Classification Unit of the Office for National Statistics, undergoes revisions every 10 years, with the latest being the third revision, SOC 2020, which has an unchanged conceptual basis since its inception (Office for National Statistics 2024). Thus, the current version of this classification is SOC 2020, a revised version of SOC 2010. The entity designated for classification using the Standard Occupational Classification (SOC) is again the concept of a job. Defined as “a collection of tasks or responsibilities to be performed by an individual, the idea of a job represents a fundamental aspect of the employment arrangement” (Office for National Statistics 2024). Typically organised by employers (or by the individual in the case of self-employment), the definition of jobs may be regulated by various entities such as professional organisations, employer or worker associations, and governments. Jobs are primarily identified by their corresponding job titles and categorised in SOC 2020 into groups based on two dimensional criteria, level of skill and skill specialisation.

As in the ISCO-08 taxonomy, SOC 2020 comprises nine major groups. Those are divided by 26 sub-major groups, 104 minor groups, and 412 unit groups. The following lists the nine major groups of SOC 2020, characterised by the overall qualifications, training, and experience required for proficiently carrying out tasks within the occupations classified under each major group:

1. **Managers, directors and senior officials** A significant amount of knowledge and experience of the production processes and service requirements associated with the efficient functioning of organisations and businesses.
2. **Professional occupations** A degree or equivalent qualification, with some occupations requiring postgraduate qualifications and/or a formal period of experience-related training.
3. **Associate professional occupations** An associated high-level vocational qualification, often involving a substantial period of full-time training or further study. Some additional task-related training is usually provided through a formal period of induction.
4. **Administrative and secretarial occupations** A good standard of general education. Certain occupations will require further additional vocational training to a well-defined standard (e.g. office skills).
5. **Skilled trades occupations** A substantial period of training, often provided by means of a work based training programme.
6. **Caring, leisure and other service occupations** A good standard of general education. Certain occupations will require further additional vocational training, often provided by means of a work-based training programme.
7. **Sales and customer service occupations** A general education and a programme of work-based training related to sales procedures. Some occupations require additional specific technical knowledge but are included in this major group because the primary task involves selling.
8. **Process, plant and machine operatives** The knowledge and experience necessary to operate vehicles and other mobile and stationary machinery, to operate and monitor industrial plant and equipment, to assemble products from component parts according to strict rules and procedures and subject assembled parts to routine tests. Most occupations in this major group will specify a minimum standard of competence for associated tasks and will have a related period of formal training.
9. **Elementary occupations** Occupations classified at this level will usually require a minimum general level of education (i.e. that which is acquired by the end of the period of compulsory education). Some occupations at this level will also have short periods of work-related training in areas such as health and safety, food hygiene, and customer service requirements.

### A3. Pipeline for occupation detection, classification, and gender attribution in textual data

The first step in the pipeline exhibited in Sect. 3.2 focuses on detecting occupations from textual data (occupation extraction). To accomplish this, we utilise a Large Language Model (LLM), through a zero-shot prompting approach. The LLM is tasked with scanning the text and identifying any references to occupations along with their contextual usage. The model not only identifies occupational terms but also provides corresponding descriptions, aiding in distinguishing between similarly named professions and reducing potential errors, such as hallucinations.

After detecting the occupations, the subsequent step is to associate them with the international standard classification of occupations, namely ISCO-08. To ensure consistency and comparability of the occupational terms, we develop a Linking module that connects the extracted occupations to the KG, based on the ISCO-08 taxonomy. The linking process is framed as a retrieval problem, where the LLM-generated descriptions of the detected occupations are matched to the corresponding entries in the KG. To accomplish this, both the LLM-generated descriptions and the predefined descriptions in the KG are transformed into vector embeddings. Using cosine similarity as the distance metric, we compare these embeddings in a latent space to find the closest match. By setting a similarity threshold, we effectively filter out incorrect matches, ensuring that only valid and relevant occupations are included. This process not only addresses hallucinations from the detection stage but also maintains a high level of accuracy when incorporating the data into the KG. This linking step is essential for facilitating consistent analysis and allowing us to leverage standardised occupational classifications for statistical analysis and comparison across various texts and contexts.

The final and most challenging component of the pipeline is the Gender Identification module, which aims to determine the gender associated with each detected occupation. Gender identification is crucial for compiling accurate gender statistics from the text, and it is handled through a three-step process:

- **Gendered Occupation Terms** In languages where occupations are explicitly gendered (such as grammatical gender in Greek or Spanish, as well as in some cases in English), the occupation word itself may indicate gender. For instance, in Greek, “δάσκαλος” (male teacher) and “δασκάλα” (female teacher) directly signal the gender of the individual holding the occupation. This also holds in some cases in English (e.g. actor/actress, waiter/waitress, etc.), where gender can be identified by the occupation word itself. In this case, linguistic tools such as SpaCy^4^ are utilised to automatically identify gendered terms.
- **Gendered Pronouns** When the occupation title itself does not indicate gender, we check for pronouns that may directly reference the occupation. For example, in the sentence, “He is a teacher”, the pronoun “He” specifies that the teacher is male. To identify such relationships, we construct a syntactic dependency tree using *SpaCy* to detect direct links between gendered pronouns and occupational terms.
- **Coreference Resolution** If neither the occupation word nor pronouns indicate gender, we employ coreference resolution to infer gender from the broader text. This technique identifies instances where different parts of the text refer to the same entity, such as when a pronoun later in the text clarifies the gender of an occupation mentioned earlier. For example, “The teacher began reading. His voice was loud and clear.” allows us to infer that “teacher” refers to a male due to the pronoun in the second sentence. We utilise the *Coreferee*^5^ library to implement this step, ensuring that occupations are accurately linked to their gender references.

If no clear gender indication is identified after these three steps, the occupation is excluded from the gender statistics to avoid the inclusion of inaccurate data.

Upon completing these stages, the resulting gender statistics for each occupation are incorporated into the developed KG (see subSect. 2.3). This integrated approach allows us to enrich the KG with valuable data on gender representation in textual corpora, providing a detailed breakdown of male and female participation across various occupations.

### A4. Male, female, and balanced occupations in the UK, France, and Greece (2023)

See Figs. 7, 8, 9, 10, 11, and 12.

## Footnotes

1 Underlined by the author(s). To give another example of striking bias manifested in formal settings: the Law 4604/2019 on the “Promotion of the substantial gender equality, prevention and combating gender-based violence”, provides in the article 9 for the establishment of a National Council for Gender Equality; among the members of this Council the law refers to the General Secretary for Gender Equality, using only the masculine form, despite the fact that since the establishment of the General Secretariat, the role of General Secretary has always been occupied by women.

2 A full description of ISCO-08 can be found at https://ilostat.ilo.org/resources/concepts-and-definitions/classification-occupation/.

3 Medical doctors (physicians) study, diagnose, treat and prevent illness, disease, injury, and other physical and mental impairments in humans through the application of the principles and procedures of modern medicine. They plan, supervise and evaluate the implementation of care and treatment plans by other health care providers, and conduct medical education and research activities.

## References

[CR1] Agars, M.D.: Reconsidering the impact of gender stereotypes on the advancement of women in organizations. Psychol. Women q. **28**(2), 103–111 (2004)

[CR2] Barocas, S., Hardt, M., Narayanan, A.: Fairness and machine learning: limitations and opportunities. The MIT press, Cambridge, Massachusetts, London (2023)

[CR3] Binns, R.: Fairness in machine learning: lessons from political philosophy. In: Conference on fairness, accountability and transparency, PMLR, pp. 149–159 (2018)

[CR4] Blakemore, J.E.O.: Children’s beliefs about violating gender norms: Boys shouldn’t look like girls, and girls shouldn’t act like boys. Sex Roles **48**(9), 411–419 (2003)

[CR5] Bojar, O., Buck, C., Federmann, C., Haddow, B., Koehn, P., Leveling, J., Monz, C., Pecina, P., Post, M., Saint-Amand, H., Soricut, R., Specia, L., Tamchyna, A.: Findings of the 2014 workshop on statistical machine translation. In: Proceedings of the Ninth Workshop on Statistical Machine Translation, pp. 12–58, Baltimore, Maryland, USA. Association for Computational Linguistics. https://aclanthology.org/W14-3302/ (2014). Accessed 4 Oct 2024

[CR6] Bolukbasi, T., Chang, K.-W., Zou, J.Y., Saligrama, V., Kalai, A.T.: Man is to computer programmer as woman is to homemaker? debiasing word embeddings. In: Lee, D., Sugiyama, M., Luxburg, U., Guyon, I., Garnett, R. (eds.) Advances in Neural Information Processing Systems, vol. 29, pp. 4349–4357. Curran Associates Inc, Barcelona (2016)

[CR7] Broverman, I.K., Vogel, S.R., Broverman, D.M., Clarkson, F.E., Rosenkrantz, P.S.: Sex-role stereotypes: a current appraisal. J. Soc. Issues **28**(2), 59–78 (1972)

[CR8] Caliskan, A., Bryson, J.J., Narayanan, A.: Semantics derived automatically from language corpora contain human-like biases. Science **356**(6334), 183–186 (2017). 10.1126/science.aal4230 28408601 10.1126/science.aal4230

[CR9] Carlsen, L., Bruggemann, R., Fattore, M.: Factors determining the degree of gender equality within the European Union. Qual. Quant. **57**, 1483–1499 (2023). 10.1007/s11135-022-01405-w

[CR10] Corcoran, M.E., Courant, P.N.: Sex role socialization and labor market outcomes. Am. Econ. Rev. **75**(2), 275–278 (1985)

[CR11] Crompton, R., Harris, F.: Explaining women’s employment patterns: orientations to work revisited. Br. J. Sociol.sociol. **49**(1), 118–136 (1998) 9569774

[CR12] De Gioannis, E.: On the association between gender-science stereotypes’ endorsement and gender bias attribution. Qual. Quant. **58**, 3087–3106 (2024). 10.1007/s11135-023-01790-w

[CR13] Ducel, F., Névéol, A., Fort, K.: “You’ll be a nurse, my son!” automatically assessing gender biases in autoregressive language models in French and Italian. Lang. Resour. Eval.resour. Eval. (2024). 10.1007/s10579-024-09780-6

[CR14] Duncan, O.D., Duncan, B.: A methodological analysis of segregation indexes. Am. Sociol. Rev. **20**(2), 210–217 (1955). 10.2307/2088328

[CR15] Eagly, A.H.: Sex differences in social behavior: a social-role interpretation. Lawrence Erlbaum Associates, Hillsdale, NJ (1987)

[CR16] Eagly, A.H., Karau, S.J.: Role congruity theory of prejudice toward female leaders. Psychol. Rev. **109**(3), 573–598 (2002) 12088246 10.1037/0033-295x.109.3.573

[CR17] Eagly, A.H., Nater, C., Miller, D.I., Kaufmann, M., Sczesny, S.: Gender stereotypes have changed: a cross-temporal meta-analysis of U.S. public opinion polls from 1946 to 2018. Am. Psychol. **75**(3), 301–315 (2020) 31318237 10.1037/amp0000494

[CR18] EIGE—European Institute for Gender Equality Glossary and Thesaurus: ISSN: 2467–3692, Catalogue number (Publications Office): MH-AC-16–001-D5-I. https://eige.europa.eu/publications-resources/thesaurus/overview (2024). Accessed 21 Sept 2024

[CR19] England, P., Herbert, M.S., Kilbourne, B.S., Reid, L.L., Megdal, L.M.: The gendered valuation of occupations and skills: earnings in 1980 census occupations. Soc. Forces **73**(1), 65–100 (1994)

[CR20] EU Science Hub: Gender gaps in education and employment. https://joint-research-centre.ec.europa.eu/scientific-activities-z/gender-gaps-education-and-employment (2024). Accessed 19 Sept 2024

[CR21] European Commission: Advisory Committee on Equal Opportunities for Women and Men: Opinion on “Breaking gender stereotypes in the media”. https://violenciagenero.igualdad.gob.es/wp-content/uploads/2010_12_Opinion_Breaking_Gender_Stereotypes12.pdf. (2010). Accessed 19 Sept 2024

[CR22] European Commission: Communication from the Commission to the European Parliament, the Council, the European Economic and Social Committee of the Regions, A Union of Equality: Gender Equality Strategy 2020–2025. https://op.europa.eu/en/publication-detail/-/publication/4ed128c0-5ec5-11ea-b735-01aa75ed71a1/language-en (2020). Accessed 10 Sept 2024

[CR23] European Commission: Leveraging Artificial Intelligence to maintain the ESCO Occupations Pillar, EU Directorate-General for Employment, social affairs and inclusion. https://esco.ec.europa.eu/system/files/2022-03/leveragingArtificialIntelligenceToMaintainTheESCOOccupationsPillarReport%20%281%29.pdf (2021). Accessed 19 Sept 2024

[CR24] EUROSTAT: European Union Labour Force Survey Database User Guide. https://www.gesis.org/missy/files/documents/EU-LFS/eu-labour-force-survey-database-user-guide.pdf (2024). Accessed 12 Sept 2024

[CR25] Ferragina, E., Deeming, C.: Comparative mainstreaming? mapping the uses of the comparative method in social policy, sociology and political science since the 1970s. JESP **33**(1), 132–147 (2023). 10.1177/09589287221128438

[CR26] Fox, L., Barth, J.M.: Gender-typed occupational aspirations among young children: the role of parents and teachers. Sex Roles **77**(5–6), 277–290 (2017)

[CR27] Gaskell, J.: Conceptions of skill and the work of women: Some historical and political issues. Atlantis-Crit. Stud. G. **8**(2), 11–26 (1983)

[CR28] Ghosh, S., Caliskan, A.: ChatGPT perpetuates gender bias in machine translation and ignores non-gendered pronouns: Findings across Bengali and five other low-resource languages. In: Proceedings of the 2023 AAAI/ACM, Conference on AI, Ethics, and Society, pp. 901–912 (2023)

[CR29] Glick, P., Wilk, K., Perreault, M.: Images of occupations: components of gender and status in occupational stereotypes. Sex Roles **32**(9–10), 565–582 (1995)

[CR30] Gorti, A., Gaur, M., Chadha, A. Unboxing Occupational Bias: Grounded Debiasing LLMs with US Labor Data. arXiv preprint. https://arxiv.org/abs/2408.11247 (2024). Accessed 15 Sept 2024

[CR31] Gottfredson, L.S.: Circumscription and compromise: a developmental theory of occupational aspirations. J. Couns. Psychol.couns. Psychol. **28**(6), 545–579 (1981)

[CR32] Greenough, W.T., Black, J.E., Wallace, C.S.: Experience and brain development. Child Dev. **58**, 539–559 (1987) 3038480

[CR33] Heilman, M.E.: Description and prescription: how gender stereotypes prevent women’s ascent up the organizational ladder. J. Soc. Issues **57**(4), 657–674 (2001)

[CR34] Heilman, M.E.: Gender stereotypes and workplace bias. Res. Organ. Behav. **32**, 113–135 (2012)

[CR61] Heilman, M., Caleo, S., Manzi, F.: Women at Work: Pathways from Gender Stereotypes to Gender Bias and Discrimination. Annu. Rev. Organ. Psychol. Organ. Behav. **11**(1), 165–192 (2024). 10.1146/annurev-orgpsych-110721-034105

[CR35] Hogan, A., Blomqvist, E., Cochez, M., d’Amato, C., de Melo, G., Gutiérrez, C., Zimmermann, A.: Knowledge graphs. Acm Comput. Surv. **54**(4), 1–37 (2021). 10.1145/3447772

[CR36] ILO: International Standard Classification of Occupations ISCO-08, Volume I, Structure, group definitions and correspondence tables. International Labour Office, Geneva (2012)

[CR37] ILO: The International Standard Classification of Occupations (ISCO-08) companion guide. International Labour Office, Geneva (2023a)

[CR38] ILO: The International Standard Classification of Occupations (ISCO-08): Recent developments and revision. International Labour Office, Geneva (2023b)

[CR39] Khan, H.U.R., Khan, A., Zaman, K., et al.: Gender discrimination in education, health, and labour market: a voice for equality. Qual. Quant. **51**, 2245–2266 (2017). 10.1007/s11135-016-0384-4

[CR40] Kirk, H.R., Jun, Y., Volpin, F., Iqbal, H., Benussi, E., Dreyer, F., Shtedritski, A., Asano, Y.: Bias out-of-the-box: an empirical analysis of intersectional occupational biases in popular generative language models. Adv. Neural. Inf. Process. Syst. **34**, 2611–2624 (2021)

[CR41] Leavy, S., Meaney, G., Wade, K., Greene, D.: Mitigating gender bias in machine learning data sets. In: Bias and Social Aspects in Search and Recommendation—First International Workshop BIAS 2020, Proceedings 1, Lisbon, Portugal, pp. 12–26. Springer International Publishing (2020)

[CR42] Macrae, C.N., Milne, A.B., Bodenhausen, G.V.: Stereotypes as energy-saving devices: a peek inside the cognitive toolbox. J. Pers. Soc. Psychol. **66**(1), 37–47 (1994)

[CR43] Mehrabi, N., Morstatter, F., Saxena, N., Lerman, K., Galstyan, A.: A survey on bias and fairness in machine learning. ACM Comput. Surv.comput. Surv. **54**(6), 1–35 (2021)

[CR44] Nosek, B.A., Smyth, F.L., Sriram, N., Lindner, N.M., Devos, T., Ayala, A., Greenwald, A.G.: National differences in gender–science stereotypes predict national sex differences in science and math achievement. Proc. Natl. Acad. Sci. U. S. a. **106**(26), 10593–10597 (2009) 19549876 10.1073/pnas.0809921106 PMC2705538

[CR45] Noy, N.F., Gao, Y., Jain, A., Narayanan, A., Patterson, A., Taylor, J.: Industry-scale knowledge graphs: lessons and challenges. ACMQ **17**(2), 36–43 (2019)

[CR46] OECD: Education at a Glance 2023: OECD Indicators. OECD Publishing, Paris. 10.1787/e13bef63-en (2023). Accessed 19 Sept 2024

[CR47] Office for National Statistics: SOC 2020 Volume 1: structure and descriptions of unit groups. https://www.ons.gov.uk/methodology/classificationsandstandards/standardoccupationalclassificationsoc/soc2020/soc2020volume1structureanddescriptionsofunitgroups#principles-and-concepts (2024). Accessed 10 Sept 2024

[CR48] Pan, J., Razniewski, S., Kalo, J.C., Singhania, S., Chen, J., Dietze, S., Jabeen, H., Omeliyanenko, J., Zhang, W., Lissandrini, M., Biswas, R., de Melo, G., Bonifati, A., Vakaj, E., Dragoni, M., Graux, D.: Large Language Models and Knowledge Graphs: Opportunities and Challenges. Transactions on Graph Data and Knowledge. arXiv preprint. 10.48550/arXiv.2308.06374 (2023). Accessed 10 Sept 2024

[CR49] Prates, M.O.R., Avelar, P.H., Lamb, L.C.: Assessing gender bias in machine translation: a case study with google translate. Neural Comput. Appl.comput. Appl. **32**, 6363–6381 (2020)

[CR50] Raffel, C., Shazeer, N., Roberts, A., Lee, K., Narang, S., Matena, M., Liu, P.J.: Exploring the limits of transfer learning with a unified text-to-text transformer. JMLR **21**(140), 1–67 (2020)34305477

[CR51] Reyna, C.: Lazy, dumb, or industrious: when stereotypes convey attribution information in the classroom. Educ. Psychol. Rev. **12**, 85–110 (2000)

[CR52] Salinas, A., Shah, P., Huang, Y., McCormack, R., Morstatter, F.: The unequal opportunities of large language models: examining demographic biases in job recommendations by chatgpt and llama. In: Proceedings of the 3rd ACM Conference on Equity and Access in Algorithms, Mechanisms, and Optimization. EAAMO ‘23, pp. 1–15. Association for Computing Machinery, New York, NY, USA (2023). 10.1145/3617694.3623257

[CR53] Savoldi, B., Gaido, M., Bentivogli, L., Negri, M., Turchi, M.: Gender bias in machine translation. Trans. Assoc. Comput. Linguist. **9**, 845–874 (2021)

[CR54] Sheng, E., Chang, K.-W., Natarajan, P., Peng, N.: The woman worked as a babysitter: On biases in language generation. In: Proceedings of the 2019 Conference on EMNLP and the 9th International Joint Conference on Natural Language Processing (EMNLP-IJCNLP), pp. 3407–3412. ACL, Hong Kong, China (2019). https://aclanthology.org/D19-1339

[CR55] Symeonaki, M., Filopoulou, C.: Quantifying gender distances in education, occupation and unemployment. Equal Divers Incl. **36**(4), 340–361 (2017). 10.1108/EDI-11-2016-0106

[CR56] Symeonaki, M., Karamessini, M., Stamatopoulou, G.: Gender-based differences in the impact of the economic crisis on labour market flows in Southern Europe. In: Bozeman, J., Skiadas, C. (eds.) Data Analysis and Applications: New and Classical Approaches, pp. 107–120. ISTE Science Publishing, London (2019)

[CR57] Thakur, V.: Unveiling Gender Bias in Terms of Profession Across LLMs: Analyzing and Addressing Sociological Implications. arXiv preprints. https://arxiv.org/abs/2307.09162 (2023). Accessed 15 Sept 2024.

[CR58] Vanmassenhove, E.: Gender Bias in Machine Translation and the Era of Large Language Models. arXiv preprints. 10.48550/arXiv.2401.10016 (2024). Accessed 20 Sept 2024

[CR59] Welle, B., Heilman, M.E.: Formal and informal discrimination against women at work. Res. Soc. Issues Manag. **5**, 229–252 (2007)

[CR60] Zhao, J., Wang, T., Yatskar, M., Ordonez, V., Chang, K.W.: Men also like shopping: Reducing gender bias amplification using corpus level constraints. arXiv preprint. 10.48550/arXiv.1707.09457 (2017). Accessed 19 Sept 2024

